# The lived experience of breathlessness and its implications for care: a qualitative comparison in cancer, COPD, heart failure and MND

**DOI:** 10.1186/1472-684X-10-15

**Published:** 2011-10-17

**Authors:** Marjolein H Gysels, Irene J Higginson

**Affiliations:** 1 King's College London, Department of Palliative Care, Policy & Rehabilitation School of Medicine, London, UK; 2 Barcelona Centre for International Health Research (CRESIB), Barcelona, Spain

## Abstract

**Background:**

Breathlessness is one of the core symptoms, particularly persistent and frequent, towards the end of life. There is no evidence of how the experience of breathlessness differs across conditions. This paper compares the experience of breathlessness in cancer, COPD, heart failure and MND, four conditions sharing heavy symptom burdens, poor prognoses, high breathlessness rates and palliative care needs.

**Methods:**

For this qualitative study a purposive sample of 48 patients was included with a diagnosis of cancer (10), COPD (18), heart failure (10) or MND (10) and experiencing daily problems of breathlessness. Patients were recruited from the respective clinics at the hospital; specialist nurses' ward rounds and consultations, and "Breathe Easy" service users meetings in the community. Data were collected through semi-structured, in-depth interviews and participant observation. Breathlessness was compared according to six components derived from explanatory models and symptom schemata, first within groups and then across groups. Frequency counts were conducted to check the qualitative findings.

**Results:**

All conditions shared the disabling effects of breathlessness. However there were differences between the four conditions, in the specific constraints of the illness and patients' experiences with the health care context and social environment. In cancer, breathlessness signalled the (possible) presence of cancer, and functioned as a reminder of patients' mortality despite the hopes they put in surgery, therapies and new drugs. For COPD patients, breathlessness was perceived as a self-inflicted symptom. Its insidious nature and response from services disaffirmed their experience and gradually led to greater disability in the course of illness. Patients with heart failure perceived breathlessness as a contributing factor to the negative effects of other symptoms. In MND breathlessness meant that the illness was a dangerous threat to patients' lives. COPD and heart failure had similar experiences.

**Conclusion:**

Integrated palliative care is needed, that makes use of all appropriate therapeutic options, collaborative efforts from health, social care professionals, patients and caregivers, and therapies that acknowledge the dynamic interrelation of the body, mind and spirit.

## Background

A recent comparison of symptom prevalence among people with five advanced, life-limiting diseases found that breathlessness is one of the core symptoms which are particularly persistent and frequent towards the end of life [1]. Breathlessness is a symptom which affects patients with many conditions: it is a common problem in advanced cancer with a prevalence of up to 70%, 95% in chronic obstructive pulmonary disease (COPD), 88% in cardiac failure and 85% in motor neuron disease (MND) [1,2].

Breathlessness is defined by the American Thoracic Society as "a subjective experience of breathing discomfort that consists of qualitatively distinct sensations that vary in intensity" [3]. This definition appreciates the complexity of this symptom coming from interactions among multiple physiological, psychological, social and environmental factors, modulating both the quality and the intensity of the person's perception of breathlessness [4], and possibly causing secondary physiological and behavioural responses [3].

Progress in management of breathlessness has been slow. Work has focused on its clinical management and less is known about its experience [5]. Qualitative studies can provide in-depth information about effects of breathlessness on patients' and carers' lives [6]. Patients construct explanatory models (EM) to make sense of specific symptom episodes in the direct context of their illness and the broader context of the patient's life [7,8]. It is essential to take these into account, in relation to breathlessness which is difficult to manage and therefore often underestimated, insufficiently elicited or reported, and undertreated [9-11].

Even when managed by specialists, breathlessness is difficult to control [12]. It is therefore worthwhile to explore breathlessness outside of specialist knowledge and look whether its experiences are similar or different across conditions. What are the differences caused by, if there are any? Are there transferable strategies to cope with this symptom in approaches developed in other conditions?

We aimed to explore and compare the lived experience of breathlessness for patients with four conditions -COPD, heart failure, cancer and MND- all with heavy symptom burden, poor prognoses, high breathlessness rates and palliative care needs. COPD, heart failure and cancer are the most common chronic diseases worldwide and their prevalence is rising [13]. MND represents the experience of neurological disorders which need complex and intensive care from a range of health and social services [14]. Chronic illness imposes costs at all levels of society [15]. Addressing a distressing symptom as breathlessness may help considerably in the containment of the economic burden of such conditions, and improve quality of life.

## Methods

### Design

Qualitative study of face-to-face cross-sectional interviews and participant observation with patients with one of four conditions.

### Context and sample

Data were collected in a qualitative study exploring the experiences of breathlessness. The study took place in a large teaching hospital and in the community, in London.

We used a purposive sample of patients with a diagnosis of cancer, COPD, heart failure and MND and experiencing daily problems of breathlessness. Patients were recruited over eight months from respiratory, cancer, heart failure, MND clinics at the hospital; specialist nurses' ward rounds and consultations, "Breathe Easy" service users meetings, and the community. Patients were prioritised who were in the advanced stages of illness (moderate to severe breathlessness in COPD, people with New York Heart Association stage 3 or 4 disease in heart failure, patients with palliative care needs in cancer and MND). We asked patients to participate who were able and willing to communicate their experiences in a lengthy interview, in order to maximise understanding of how they experience breathlessness and care towards this symptom.

### Data collection

Consenting patients were interviewed at home, except for two patients, who preferred to be interviewed in the researcher's office. The interviews were in-depth and semi-structured; a topic guide was used as an aide-memoir, following a number of broad themes, ensuring that all the relevant topics were covered. All were tape recorded and transcribed verbatim, except two, which could not be recorded for technical reasons. During these, detailed notes were taken and written up immediately after the interview. Discussions with participants which were not recorded, e.g. what they told the researcher after the interview, when the tape recorder had already been switched off, and the participant took up one of the topics again from the interview, were also written down afterwards.

The researcher was a participant observer in consultations of health professionals and in Breathe Easy meetings. Field notes were kept of non-verbal aspects (e.g. body language or tone of voice) and in situations in which it was inappropriate to tape record, which provided additional data to the more theoretical insights being generated. Data were collected until a point of saturation was reached, i.e. when no more new information emerged from the data, and no additional insights were generated.

### Analysis

We used the concept of EM [7] and Symptom Schema [16] as the framework for the analysis of the patients' experiences of breathlessness. We constructed a coding scheme relevant for the symptom of breathlessness by combining components of these models that were: label, cause of illness, nature of breathlessness, time line, treatment/coping (separately for breathlessness and for illness), consequences of breathlessness in the context of illness. The texts of transcribed interviews and discussions were read and coded in NVivo. Initial detailed coding allowed the identification of the range of patients' perceptions or experiences within these components and the discovery of unexpected dimensions. Through a process of constantly integrating categories and their properties, or constant comparison, the findings became relevant at a more abstract level. In parallel, meaningful terms and phrases relating to the components of the coding scheme were categorised in an ExceL spread sheet, to enhance the visibility of similarities and differences between cases. The links between condition and the more abstract categories generated in the analysis were checked by frequency counts. These were presented in a table to show the numerical distribution across the different conditions (see Table 1). The analysis was carried out in several stages. First, we focused on within-group experiences and perspectives in relation to the components of EMs. Second, we compared and contrasted experiences in different conditions. Our comparative approach was case-based and adopted an interpretive model which starts from the idea that any behavior, perception, attitude can only be understood within its social context.

**Table 1:** Themes in conditions

THEMES in the experience of breathlessness		CONDITIONS compared			
**Themes**	**Sub-themes**	**Cancer**	**COPD**	**HF**	**MND**

Nature	Manifestation (sudden/surreptitious)	4	12	7	10

	Evaluative (worst symptom)	1	11	4	2

	Associated feelings (fear)	4	18	2	10

	Influence on life (inhibiting)	3	17	9	10

Label	No immediate diagnosis	1	18		

	Diagnosis carrying meaning	10	1	10	

	Diagnosis not carrying meaning		17		

	Long search for diagnosis				10

Time line	Awareness of diagnosis after test for symptoms	10			

	Awareness of diagnosis in retrospect		18		

	Awareness after manifestation of symptoms and confirmed by test			10	2

	Step by step awareness				8

Cause	Internal	2		7	

	External	5	18		

	Both	2	2	3	

	Not clear	1			10

Coping symptom	No advice	9	12	8	

	PR	1	5	2	

	Essential breathing aids				10

Coping illness	Psychological/spiritual mechanisms	7	4	6	10

	Self-management		5		

	Caution strategies		11	10	

	Hope in surgery for cure	4		7	

	Hope in treatment for extending life	5			

	Lack of coping (suffering)		11	2	

	Reliance on medication			10	

Consequences	Family	3	9	3	10

	Financial	2	12	2	3

	Disability	2	18	10	10

	Search for cure	3			

	Fear of dying/death	3	12	6	

	Stigma	2	10	3	3

	Access to care		12	6	

	Copers		5		

The numbers are representative of how many times something is mentioned throughout the interviews, therefore they may not always add up to the number of interviewees in each group.

Accuracy of the data was verified in various ways. First, the data from the interviews were confronted with data from participant observation in consultations between health professionals and patients. Frequency counts were carried out which showed clear patterns in occurrence matching those found in the interviews. We also present direct illustrative quotations from the data.

## Results

We conducted a total of 48 interviews with patients. Interviews lasted between 40 and 150 minutes, with a mean of 65 minutes (see Table 2).

**Table 2:** Patient details

Types of participants	Recruitment setting	Patients interviewed	Age patient	Gender patient	Number and relation of patients' carers
Patients with cancer	Outpatient clinic	10	Range: 52-84 Median: 65	5 women 5 men	3 spouses 1 daughter

Patients with COPD	Outpatient clinic, service users meetings, and community	18	Range: 52-78 Median: 69	9 women 5 men	6 spouses 1 daughter 1 sister

Patients with cardiac failure	Outpatient clinic	10	Range: 61-80 Median: 69	3 women 7 men	3 spouses

MND patients	Outpatient clinic	10	Range: 24-77 Median: 42	1 woman 9 men	8 spouses 1 mother

### Cancer

#### The nature of breathlessness

Patients described breathlessness in terms of how it manifested itself and the feelings it evoked. It appeared suddenly and it was described as frightening. Those people who said it was untypical for them to panic said that breathlessness had this effect on them. Three patients said that breathlessness had an inhibiting effect, which made them more cautious in what they undertook, and it made them worry about the future.

#### Label

A diagnosis of cancer was preceded by tests, not always giving an immediate definite result, requiring more tests to be carried out over a period of time. This was important in the case of a diagnosis of cancer as it was loaded with meaning.

'*I think when they say that word it makes you like ill anyway don't ya?'*

#### Timeline

To receive the label 'cancer' was described as a shock by all patients and immediately associated with dying. The concern about the breathing problems for which they originally sought professional help, faded in importance when cancer was suspected. In retrospect, the symptoms were experienced as announcing cancer. To experience this first sudden sensation of breathlessness was described as 'strange', 'a mystery'.

When they received their diagnosis they either enquired about or were given a prognosis.

'...and er they said it's cancer and I asked him straight I said well: what's the life expectancy?'

#### Cause

Most of the patients mentioned an external cause for their diagnosis (7), exclusively or in combination with an internal cause. External causes were influences from outside, such as smoking, a 'bad pattern of life', or environmental, due to unhealthy work places, or stressful experiences. Internal causes were due to a malfunctioning in the body.

#### Treatment/coping with breathlessness

Nine out of the 10 patients said they had never received advice on how to deal with breathlessness. Only one patient was referred to pulmonary rehabilitation (PR) where she learned breathing techniques and other strategies to manage breathlessness. Doctors prescribed inhalers but patients felt they had not explained how to use them. Patients said they were useless.

'I was taking that [inhaler] on the way, [...] and I was stopping in the street taking that and it never done a thing. I was breathing in for dear life but it wasn't helping. [...] I think that if that don't work it makes the panic worse, you're in a state because you've got nothing else to take.'

One patient discovered that his breathing improved when taking the morphine tablets which he had been prescribed for pain. One patient received oxygen at home. Self-management consisted of pacing and goal setting, distraction strategies, and one patient always carried a cardboard bag.

#### Treatment/coping with cancer

Contact with health professionals revolved around tests, and treatment decisions. When ambivalent results came back they had the tests repeated to be certain about whether the symptoms were due to cancer. This was a stressful time for patients as it involved collaboration with painful procedures and uncertainty. Patients underwent surgery with the expectation for cure. Those who could not be operated went through treatment in the hope to get cured.

Psychological and spiritual coping mechanisms were most often mentioned to cope with a poor prognosis. Where this was too difficult to face, denial, humour, showing strength, spirituality, and maintaining normality were mentioned. There were two cases of limiting one's view to the next doctor's appointment. One person wanted to put the experience behind him after having had a successful operation.

#### Consequences

Patients mentioned a variety of consequences of breathlessness as part of a cancer diagnosis. They felt guilty for unhealthy lifestyles and blamed themselves for this. They put their trust in medicine and they hoped for cure through surgery.

'I'd hope to have a lung transplant'

Those who had been told they could not have an operation hoped that treatment would give them a bit longer to live. At the same time they were aware about the progressiveness of their condition and they expressed concerns about the future. They felt a diminishing quality of life due to worsening symptoms. They were worried about losing their independence and becoming a burden to their family.

### COPD

#### The nature of breathlessness

In the beginning breathlessness showed itself as hardly noticeable and it developed gradually. Patients easily adapted to its restrictions as they accepted them silently in everyday routines and conveniently explained them away.

"...It (breathlessness) is over time slowing down everything, and it wasn't until my daughter said: 'You're getting so slow mum you're like an old lady.' [...] I suppose that was when I became aware that it was having an effect on my everyday life."

Patients spoke about breathlessness in terms of the feelings associated with bad episodes: distress, anxiety, panic and fear of dying. They also told as frequently about the disability it caused, inhibiting every movement. Eleven of the 18 patients evaluated it as their worst symptom.

#### Label

Patients only consulted health professionals after having lived for long periods with breathlessness. When they were confronted with a crisis, they were alarmed and they sought medical help. COPD derived its meaning from other diseases. Those with a smoking history feared to receive the news they had cancer.

"I've been to the doctors and they've told me I've got COPD what is it? Now the doctor hasn't even explained what those 4 letters mean and the worst thing about it is, it starts off with C, and first thing you think: Oh have I got cancer but this is the thing that is wrong its not a well known disease yet its one of the greatest killers."

Instead of a definite diagnosis, patients received different labels: bronchitis, COPD, asthma, emphysema. The acronym of COPD starts with "chronic", implying a long-term and relatively manageable condition.

#### Timeline

Over time, sufferers found out for themselves what breathlessness means: daily troubles with breathing, impaired mobility, anxiety restricting them to home, growing dependence. Patients started a quest for effective treatment once they realised the full extent of the symptoms. However, this proved to deliver little in terms of relief. Over time they built up knowledge, through personal experience, often without much expert advice. In the advanced stages of illness they re-evaluated the diagnosis and identified issues which had been left unaddressed. Patients found that full disclosure of the nature and course of breathlessness was crucial for its management.

"At the time if I can remember rightly we weren't really told that [...] it would gradually get worse. [...] We were just told this is what you've got, get on with it."

#### Cause

All 18 patients with COPD mentioned external causes for their diagnosis, one person added an internal cause. All patients attributed breathlessness to a lifetime of smoking. They mentioned its detrimental effects:

"I am poisoned."

"...it's a terrible condition but then it's self-inflicted with the cigarettes"

Patients felt guilty about breathlessness; they felt responsible for having it brought onto themselves.

#### Treatment/coping with breathlessness

All patients had experience with inhalers and steroids, but inhalers offered minimal relief, and steroids caused side effects. Five patients relied on oxygen at home. Six had a nebuliser, two had purchased one on their own. They were unanimous that it helped their breathing and calmed them down.

Eight patients said they had never had any advice on how to manage their breathlessness.

"I don't know what to do[...] you can't run to the doctor every 5 minutes, you can't run to casualty, you need to know how to deal with things yourself, [...] at least be discussing them..."

In the absence of professional advice one patient mentioned simple strategies: steam baths, a fan, open windows.

Five patients went to PR classes. They learned the necessary skills and behaviours to self-manage and this gave them an acceptable quality of life.

#### Treatment/coping with COPD

Only the patients who received PR said they cope reasonably well. Those who did not said that breathlessness inhibited every movement which led patients to reduce their activities even more, to the essentials. Gradual deconditioning made them realise that there is no 'magic pill' for their suffering.

#### Consequences

Disability was mentioned most frequently. They were often restricted to the home leading to social isolation. Disability caused financial hardship as they lost their jobs, partners took on the role of carer over time. They spoke of having problems with access to care. Stigma added to patients suffering. Patients were not prepared to face the future and expressed only to be able to cope with one day at a time.

### Heart failure

#### The nature of breathlessness

Patients with heart failure could sometimes not trace the first experiences of breathlessness back to a specific moment. This was due to the slow process of how it develops over time. Patients adapted their activities to what the breathlessness allowed them to do and they got used to it as a part of daily life.

"...but the breathlessness as I have now has been creeping on quite insidiously so it's hard to define."

They described the symptom mostly in terms of the limitations it imposed to their daily functioning, and less so in evaluative or emotional terms.

#### Label

Patients used the terms heart failure and heart attack to refer to what was wrong with their health. They also used more specific labels and descriptions, and spoke of cardiomyopathy, angina, the ejection fraction being lower than normal, hardening of the heart walls, arrythmia etc., using the terms with which their physicians had communicated the diagnosis. These were immediate diagnoses, except for one person where cancer was suspected. Heart failure carried meaning, patients recognised the seriousness of their condition.

#### Timeline

After the diagnosis all patients underwent surgery, except one person whose doctor told him that there was only 30% that the operation would be successful. Surgery was life-saving.

"...(the doctor said): we'll have you in and I say: 'Oh but what if I don't have the operation?' He said well: 'Your next heart attack 'll kill ya."

When time had passed after the operation, patients realised that surgery did not bring them back to their old healthy selves. They experienced several symptoms they actively had to manage: pain, the build up of fluid, tiredness, breathlessness.

#### Cause

All patients attributed their heart problems to internal causes, due to a mechanical fault in their body.

"I think it's because the heart 's not pumping as well as it should be."

Two also identified external causes, one to smoking and one to an injury at work.

#### Treatment/coping with breathlessness

All patients had experience with inhalers but found them of limited use. Only one used a nebuliser and oxygen which made him feel safe. Two patients went to PR classes. Except for those two, all others said that they had never received advice on how to cope with breathlessness.

Patients said that they did not always report breathlessness, they did not see that as the role of the clinic; health professionals never asked about breathlessness, and one patient was concerned she would give the impression of being a hypochondriac.

In the absence of professional advice they applied avoidance, planning and pacing strategies.

#### Treatment/coping with heart failure

All patients except one had accepted surgery despite the information they received about the risks involved. They talked about the hope it had given them initially, however, after the operation they had slowly realised that it had not brought them the improvements they had expected.

Carer: You sort of thought that was [...] the major thing the operation, and everything was gonna be alright after that.

Patient:... and it's a bit of an awakening although we were told beforehand. I think you tend to put too much hope.

They told about symptoms becoming worse over time and they were prescribed hefty pill regimes needing active management. Some of the medication caused heavy side-effects or created other symptoms and patients balanced the benefits versus their actual quality of life.

#### Consequences

Disability was mentioned most frequently as a consequence of heart failure.

"... sometimes it depresses me coz you feel [...] useless, incompetent, you can't do a simple task, like I try to put me socks on and I'm struggling and I might get one on with a bit of luck and I go to get the other one and I have to give up and I'm upset."

They spoke of problems with access to care, and to the slow response from social services. They complained about little coordination among health professionals in the community.

Breathlessness was a contributing factor to the negative effects of other symptoms. Stigma was mentioned three times by patients.

Patients were worried about the future, they feared they would experience another heart attack and die. Daily life with troubling symptoms and restricted mobility made them think about life before death and they expressed uncertainty if life would be worthwhile.

"...well quality of life is more important than taking beta-blockers. Erm that will protect you but make you a vegetable at the same time, erm ... I'm not interested in being a vegetable..."

### MND

#### The nature of breathlessness

Patients traced back first breathing difficulties when they, or a carer noticed a different breathing pattern had developed. This coincided with other symptoms such as disturbed sleep, nightmares, hallucinations, headaches etc. They learned that these symptoms were related to their breathing when seeking help at the hospital.

Patients described the sudden onset of breathlessness. All spoke about the anxiety and fear it went together with. They said it had worsened their disability, limiting them even more. Breathlessness made patients realise that the illness affects mechanisms essential for living.

Only two patients evaluated breathlessness as the worst symptom. Other symptoms overshadowed breathlessness such as loss of speech.

One patient made a distinction between daily and critical breathlessness. When experiencing critical breathlessness he described not being able to breathe at all and this went together with panic, it was a matter of life and death, requiring fast action.

#### Label

Patients had experienced a long search before they received a diagnosis of MND. As this is a rare condition, health practitioners outside neurology were not always familiar with the symptoms. A man who experienced speech difficulties was first referred to the dentist, another person whose foot dropped was sent to the rheumatologist. Several months, to a year passed between first symptoms and diagnosis. Two of the 10 patients found they had MND through self-diagnosis. Seven people learned about the condition when they received their diagnosis. When they were asked about the effect of knowing they had MND they used words, phrases, that showed the serious, drastic impact of this news: 'devastated', 'a bolt out of the blue', 'you turn a corner', 'death sentence'.

#### Timeline

The main message of the diagnosis in this condition was the prognosis, which is uncertain and short. This drastically changed outlook to the future was managed in different ways. When still little affected by the illness people went on holidays, and delayed or restricted disclosure.

People needed time to come to terms with symptoms and future. They became aware of this in a step-by-step manner, each time when they were affected by another loss. Their strategy was to live in the present. They acknowledged their prospect of worsening symptoms. One person said: '*this doesn't take any prisoners*' when comparing MND to cancer, another life-limiting illness where patients might be released by cure.

#### Cause

All MND patients said that the cause of the illness was unknown, even to medicine. Therefore it overcame people, which happened beyond control.

"... *er motor neurones disease it's not caused by anything that we know of they don't know, you just get it..."*

#### Treatment/coping with breathlessness

Nine patients used non-invasive intermittent positive pressure ventilation (NIPPY) and for one it was considered. Three patients reported difficulties using it. For those who had incorporated it in their daily routines it was the solution for their previous breathing problems. They were monitored by a respiratory physician at the hospital and had received advice from physiotherapists. They used positional and other techniques to relieve breathing difficulties.

Two people received medication, in the case of distressing breathlessness the person was given morphine. In the other case the carer was advised to give Lorazepam to the patient when critical breathlessness set in.

The respiratory physician at the hospital had suggested a tracheostomy to one person which he found very upsetting.

#### Treatment/coping with MND

Patients said there was a team of specialists they could always rely on. They were grateful for their help, however in times they were not needed they appreciated to be left alone. They tried to maintain normality in their lives. Also technology was accepted both with reluctance and appreciation.

One person had undergone anticipatory surgery. He expressed the difficulty to come to terms with the gastrostomy, another loss, which was not even directly brought about by the illness.

Six patients mentioned they took Riluzole, a drug aimed to slow down the progression of the illness.

They resorted in the absence of hope for a cure to psychological and spiritual coping mechanisms.

"There's nothing worse that can happen now..."

#### Consequences

All patients spoke about the effects of progressing disability: loss of work, independence, future, relationships, sexuality, one's home.

"MND turned life totally upside down"

Some patients mentioned also gains because of the illness, which were closer relationships with families and a belief in the kindness of mankind.

All patients were concerned about their illness affecting their family, relating to carers in their caregiving role or what would happen to their loved ones after their death. Patients prepared for death, if not through acceptance, through sorting out financial matters for after their death.

Three patients experienced stigma as part of MND.

## Discussion

This study found differences in how patients experience breathlessness across different conditions, as a physical sensation. These patterns, characteristic for different illnesses are further shaped by their experiences with the health care context and social environment [17-19]. This has an effect on how patients make sense of their symptoms, whether these are recognised, when these trigger a response in terms of health seeking, who is consulted, and what treatment is considered appropriate. The labelling of the condition responsible for one's symptoms frames the perception of the illness [20,21]. There appeared systematic differences in the type of causes patients ascribed to their illness across the four conditions. External causes were linked to the idea that these fell within their control and responsibility. The experience of breathlessness is dependent on the distinct treatment approaches prevailing in medical specialist cultures. In cancer, there was little attention to breathlessness within a test and cure-oriented approach. COPD patients initially experienced breathlessness as a symptom of a chronic illness. Responses from services disaffirmed patients' experience of breathlessness, discouraging them from presenting their problems [8]. Patients with heart failure described breathlessness as a contributing factor to the negative effects of other symptoms. Breathlessness in MND appeared as a constant reminder that the illness was a dangerous threat to patients' lives, undoing their attempts to maintain normality. In the face of these challenges they resorted to psychological and spiritual coping strategies.

Although we identified rather homogenous experiences in separate diseases, unique experiences within these diseases need to be recognised. The patients in this study belonged to separate conditions. In reality more patients have multiple conditions and complex patterns of symptoms. However, the EM and symptom schema provided systematic frameworks for the comparison across conditions and the approach of the constant comparative method also provided set procedures for the analysis while still respecting the evolving, inductive nature of enquiry needed for these qualitative data.

There are only a handful of studies that have compared the needs of patients with an advanced condition where breathlessness is a major symptom. They have all focused on cancer and COPD [22-27], except for Bekelman et al. [28] who compared cancer and heart failure. The studies found that patients had similar symptom burdens, and equivalent needs for palliative care, while those patients with other than cancer diagnoses had problems with access and utilisation of care. A few studies that took breathlessness as the unit of comparison in different conditions showed differences in the pattern of breathlessness [29,30], which is related to differences in disease progression [10,31]. Our study is the first one to compare breathlessness across multiple conditions. It adds to the insights of previous studies by showing how breathlessness affects patients in different ways across different conditions. Through a broad approach we obtained a contextualised view of how the symptom is experienced within the constraints the illness imposes on the body and the responses this evokes from health care and society. In this way it was possible to include all aspects that account for 'total breathlessness' [32].

The obvious conclusion on the basis of our findings that breathlessness is experienced differently in different conditions would be to refine its management and tailor interventions to specific patient groups. However, we found that the problems were often a result of the disease-specific services and treatments that patients with different diagnoses receive. Examples are the barriers to access of services for patients with COPD and heart failure, or the primacy given to disease status in the case of cancer influencing the perception and management of symptoms. Such specialist approaches are too narrow in focus for the relief of a complex symptom as breathlessness as they pay insufficient attention to what this symptom means to the daily life of those affected [33]. Palliative care is equipped to address the different dimensions -the physical, psychological and spiritual- of breathlessness with its holistic approach. This enables the recognition of the distress and disability it causes across all the conditions, and ensures equitable distribution of resources. At the same time its focus on the individual can build on the expertise developed in different conditions. This study started a much needed evidence-base of the patient's experience of breathlessness across different life-limiting illnesses. This symptom crosses disease, medical and body-mind boundaries [34]. It needs all appropriate therapeutic options from across the disease spectrum, collaborative efforts from health, social care professionals, patients and caregivers, and therapies that acknowledge the dynamic interrelation of the body, mind and spirit.

Such integrated models of care are needed for complex problems in chronic, life-limiting illness [35]. Interventions that build on broader conceptions of the symptom such as the work by Corner [36,37], and the Breathlessness Intervention Service [38] need further testing with breathless patients from several patient groups.

## Limitations of the study

The findings from this study can not be generalised to populations. This study rather aimed to tease out differences and similarities in the experience of breathlessness across the conditions focused on, however the findings permit the type of generalisations that can be made from and about cases (idiographic generalisation).

## Conclusion

This is the first study that compared breathlessness in four conditions, to find that the experience of the symptom differs across these separate conditions. These findings have important implications for practice as the problems identified were often a result of the disease-specific services and treatments that patients with different diagnoses receive. They show that integrated models of care based on collaborative efforts among all those involved and a broad conception of the symptom are needed.

## Ethics approval

Ethics approval was granted by the King's College Hospital Local Research Ethics Committee and by representative bodies (the hospital and community research and development committees) in the boroughs in which the study was carried out. The data was anonymised.

## Competing interests

The authors declare that they have no competing interests.

## Authors' contributions

MG and IJH jointly conceived and designed the study, obtained ethics approval, interpreted the data and wrote the paper. MG recruited the participants, collected the data and conducted the analysis. IJH obtained the funding, checked and reviewed the categories that emerged from the analysis. IJH and MG are the guarantors of the paper. All authors read and approved the final manuscript.

## Pre-publication history

The pre-publication history for this paper can be accessed here:

http://www.biomedcentral.com/1472-684X/10/15/prepub
